# Synthesis and Characterization of Biochar Obtained by Partial Delignification of Waste Biomass

**DOI:** 10.3390/molecules30234505

**Published:** 2025-11-22

**Authors:** Gabriel Vasilievici, Mia Sanda, Marian Băjan, Cristina Dușescu-Vasile, Ion Onuțu, Gheorghe Brănoiu, Dorin Bomboș, Abeer Baioun, Anca Florentina Borcea, Andra-Ioana Stănică

**Affiliations:** 1 National Institute for Research Development for Chemistry and Petrochemistry-ICECHIM-București, 202 Spl. Independenței, 060021 Bucharest, Romania; gvasilievici@icechim.ro; 2 Department of Petroleum Refining Engineering and Environmental Protection, Petroleum-Gas University of Ploiesti, 39 Bucharest Blvd., 100680 Ploiesti, Romania; 3 Department of Petroleum Geology and Reservoir Engineering, Petroleum-Gas University of Ploiesti, 39 Bucharest Blvd., 100680 Ploiesti, Romania; gheorghe.branoiu@upg-ploiesti.ro; 4 Chemistry Department, Petroleum-Gas University of Ploiesti, 39 Bucharest Blvd., 100680 Ploiesti, Romania; 5 Chemistry Department, Faculty of Science, University of Damascus, Damascus P.O. Box 30621, Syria; 6 Technological Highschool “Toma Socolescu”, Gheorghe Grigore Cantacuzino St., 328, 100466, Ploiești, Romania; ioanaandra@yahoo.com

**Keywords:** waste biomass, adsorption, activated biochar, grape seeds

## Abstract

Biochar activation is achieved by removing tar formed in the pores during the thermal treatment of biomass, thereby increasing pore volume. This process typically involves entraining tar with steam at elevated temperatures for extended durations. In this study, a reduction in lignin content in grape seeds through partial solubilization, prior to thermal treatment, is proposed. Initially, grape seeds were treated with a basic sodium sulfide solution, followed by conditioning with either phosphoric acid or zinc chloride and then drying and calcination. The synthesized adsorbents were characterized using scanning electron microscopy (SEM), textural analysis, Fourier transform infrared spectroscopy (FTIR), X-ray diffraction (XRD), and evaluation of toluene adsorption capacity. Textural analysis indicated that conditioning with phosphoric acid or zinc chloride increased the specific surface area of biochar by over 20%, with a higher increase observed for phosphoric acid treatment. The toluene adsorption capacity of the adsorbents was assessed in a continuous fixed-bed system. Biochar pretreated with ZnCl_2_ exhibited an adsorption capacity of 0.11 cm^3^ of toluene per gram, while biochar pretreated with phosphoric acid demonstrated a capacity of 0.14 cm^3^ per gram. These results indicate that preliminary delignification of grape seed biomass maintains its adsorption capacity of toluene at levels comparable to other adsorbents, despite a lower activation temperature.

## 1. Introduction

Activated carbon is an adsorbent with many practical uses. Carbonization and chemical activation are highly energy-intensive steps that increase the price of this adsorbent. The two steps can take place in a single step, in which the carbon impregnated with activating agents is treated at temperatures between 300 and 800 °C. Common reagents used in this process include acids, alkaline solutions, and metal salts. A highly efficient pyro-hydrochar adsorbent of lead ions from an aqueous solution was developed by Petrovic et al. They applied hydrothermal carbonization, followed by Fe/Mg metal salt impregnation and subsequent pyrolysis to grape pomace. The study demonstrates that ion exchange is enhanced by metal doping, thus creating additional active sites and intensifying ion exchange interactions. The non-uniform distribution of surface properties along with metal binding affinity contributes to the catalyst’s high capacity and selectivity [1]. Biochar, in contrast to activated carbon, is produced under milder pyrolysis conditions in an inert atmosphere using slow pyrolysis at relatively low temperatures (below 700 °C). Generally, lignin-rich feedstocks are recommended for higher biochar yields, and the carbon mass fraction, micropore volume, ash, and fixed carbon increase with increasing pyrolysis temperature [2]. Thus, biochar has emerged as a promising alternative to commercial activated carbon due to its abundant feedstocks, low cost, and reduced energy consumption. However, the main disadvantages that limit the widespread use of activated carbon in VOC reduction are pore blockage and its hygroscopic nature [3,4,5]. The blockage of pores is mainly due to the presence of lignin in the biomass. Lignin is an amorphous, polyphenolic material, being the second most abundant compound in nature after polysaccharides [6]. Due to the complex three-dimensional structure, it is not possible to determine its average molecular weight and molecular weight distribution. The carbonization processes by which ligno-cellulosic materials are transformed into coal proceed with the formation of a tar with a predominantly aromatic structure due to the contribution of the lignin component [7]. The removal of this tar by activation in the presence of steam has proven to be difficult due to the lignin precursor of the tar. Thus, to obtain a porous structure, high activation temperatures applied over long periods of time are required. Activated carbon has proven to be effective in reducing indoor pollution, being particularly versatile due to its extensive specific surface area, well-defined pore structure, and high capacity for volatile organic compounds (VOCs). This material is typically produced from a variety of carbonaceous precursors, including coal, wood, coconut shells, grape seeds, etc. Using waste materials as a feedstock helps reduce waste and supports a circular economy. This practice turns a waste product into a valuable resource, which benefits the environment [8,9]. Activated carbon can be produced in various forms, including granules, powders, or spheres, following carbonization and activation processes [10,11]. Pyrolysis is a key technique used for thermal decomposition of the dense macromolecular structures of biomass to produce fragments that can be further upgraded into value-added products [12]. Acidic characteristics of catalysts used in the pyrolysis process, particularly the distribution and strength of Brønsted and Lewis acid sites, were found to enhance the catalytic performance [13]. Zhou et al. similarly found that magnesium oxide-modified activated carbon also presents a high acetone adsorption capacity at 25 °C [14]. Recently, Akl et al. used zinc chloride-activated biochar to adsorb and eliminate different types of dyes and metal ions from wastewater. A noticeable increase in the recovery % using the ZCP catalyst can be attributed to its modification by ZnCl_2_. Also, the formation of an inflection point during the incomplete desorption process impacts the lifespan and regeneration cost of activated carbon (AC). This phenomenon is attributed to irreversible adsorption mechanisms, which may include chemisorption, adsorbate coupling, or decomposition [15]. Mabrouk et al. explored the surface assimilation of an analgesic on an activated carbon prepared by chemical activation with H_3_PO_4_ from grape seed waste. The use of activated carbon obtained from waste is considered an effective method for eliminating harmful substances, thus presenting an innovative and efficient approach. These findings prompt further research into the ability of biochar to adsorb a wide range of toxic VOCs from the atmosphere [16]. Many studies focused on the adsorption of aromatic hydrocarbon pollutants suggest the use of materials like organic wastes, activated carbon, or biochar as effective adsorbents [17]. Yang et al. explored the adsorption behavior of activated carbon derived from different raw materials, such as wood, charcoal, and coconut shell, onto toluene at 25 °C, 200 ppm, in a nitrogen atmosphere. Among the analyzed samples, wood-based activated carbon, which had the largest surface area and total pore volume, showed the highest adsorption capacity of 184 mg × g^−1^ [18]. After exhaustion, biochar doped with phosphorus or Zn can be used as a source of fertilizers or microelements for agricultural soil after steam stripping to remove traces of VOCs. Another valorization option would be to use it as a solid biofuel, either by fluidized bed combustion or by briquetting. The biochar produced by carbonization consists of disordered carbonaceous elementary crystallites presenting a rudimentary pore structure. Osman A.I. et al. studied the influence of biochar preparation technologies on energy consumption and agricultural carbon emissions. Their analysis shows that process and feedstock play a significant role in the properties and production rate of biochar [19].

In conclusion, the most important disadvantage of activated carbon-based adsorbents is the significant energy costs associated with their production, mainly due to the high temperatures and long activation times required. To mitigate these costs, both the temperature and the activation time can be reduced in the manufacturing process of activated carbon and biochar. This can be achieved either by partially removing the tar precursors—compounds generated during the thermal treatment of the carbonaceous raw materials used in production—or by increasing the volatility of these precursors during conditioning. Consequently, a lower concentration of tar precursors or an increased volatility will facilitate the creation of a well-defined porous structure at reduced temperatures and a shorter thermal treatment time. The novelty of this study is the application of a new method of conditioning lignocellulosic biomass for the preparation of activated biochar, namely, its partial delignification. Thus, by solubilizing the lignin fraction with a lower molecular weight—the fraction responsible for the formation of tar that clogs the pores of the prepared carbon—the activation costs are reduced. Due to the high lignin content, grape seeds were selected as the lignocellulosic biomass for the preparation of activated biochar. The objectives of the study were to (i) reduce the lignin content of the lignocellulosic biomass used in the synthesis of biochar, namely grape seeds; (ii) improve the porous structure of the biochar by conditioning with a Brønsted acid (phosphoric acid) or a Lewis acid (zinc chloride); and (iii) characterize the biochars obtained by determining the main characteristics (thermogravimetric analysis, textural characteristics, microstructural morphologies, X-ray diffraction, FTIR, and adsorption capacity of an atmospheric pollutant such as toluene).

## 2. Experimental Section

### 2.1. Materials

The reagents used in this study included orthophosphoric acid (ACS reagent, ≥85 wt.% in H_2_O), zinc chloride (reagent grade, ≥98%), and sodium sulfide nonahydrate, all from Aldrich-Sigma, Merck KGaA, Darmstat, Germany. The selection of grape seeds for the preparation of biochar was based on their composition. Thus, the content of lignin, a precursor of biochar, is high, and the polysaccharides that contribute to the completion of the porous structure are also found at high concentrations.

### 2.2. Synthesis of Adsorbent

**Figure 1:**
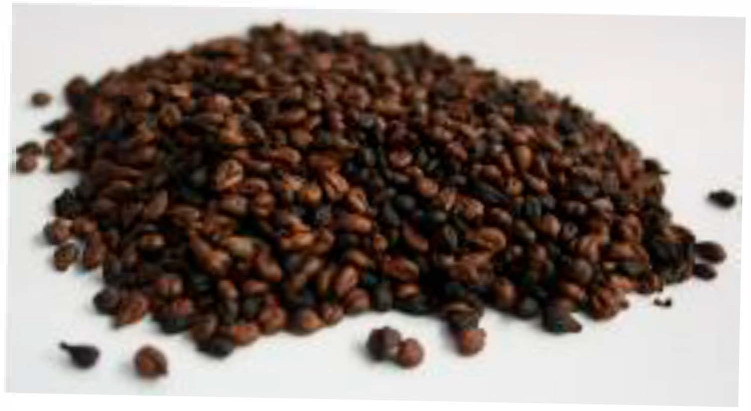
The grape seeds before treatment.

In this study, the seeds were the “Vitis vinifera Linné subsp. sativa (de Candolle) Hegi” grape variety, specifically harvested for red wine production in Boldești Scăieni, located in Prahova County, România. Following the fermentation of the must, the seeds were meticulously separated without the application of any chemical additives. They underwent a thorough washing process with distilled water until the wash water showed no signs of turbidity, ensuring the removal of impurities. Subsequently, the seeds were dried at a temperature of 105 °C for one day and were subsequently stored at ambient temperature until further analysis. The dimensions of the raw seeds ranged from 2 to 3 mm (Figure 1).

**Figure 2:**
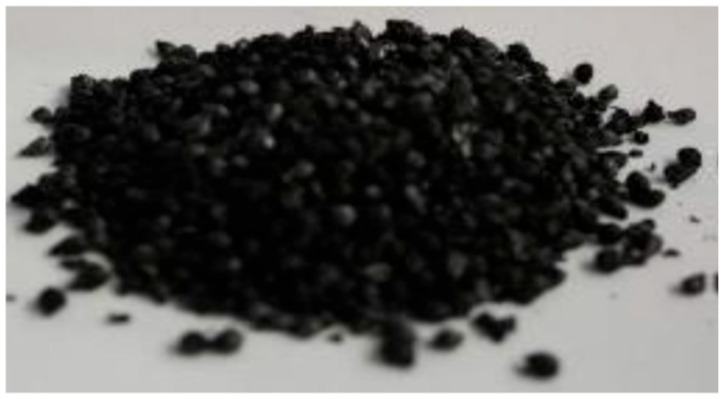
The carbon material obtained.

In the initial stage, the seeds were dried in a forced air oven at 103 °C for 4 h. Then, the grape seeds were pretreated to partially remove the lignin. For this, we applied the Kraft chemical process used in the manufacture of wood cellulose [20]. Following this, 20 g of the dried grape seeds were placed in a Parr autoclave along with 7.5 g of Na_2_S, 4.5 g of NaOH, and 138 mL of distilled water. The reaction mixture was maintained at a temperature of 170 °C and a pressure of 6.5 bar while being mechanically stirred at 125 rpm for 40 min. After the reaction, the activated carbon precursor, designated as S1, was recovered through filtration and subsequently washed with distilled water in three equal portions using a seed-to-water mass ratio of 1:5. It was then dried in an air-circulating oven at 105 °C for 6 h, resulting in a total weight of 13.8 g of activated carbon precursor (S1). Sample S1 was the control sample, which was biochar without conditioning (Figure 2).

In the next step, we performed chemical activation with phosphoric acid following a procedure similar to that reported by M.E. Fernandez et al. [21] or with zinc chloride following procedures similar to those reported by Júlia Martins Carolino et al. [22] and Magda A. Akl et al. [15]. Thus, the biochar precursor S1 was treated using the pore-filling method with aqueous solutions of the crosslinking agents phosphoric acid and zinc chloride (S2 and S3). The conditioning agents were first dissolved in distilled water and then contacted with the biochar precursor at a mass ratio of 1:5 for phosphoric acid and 1:8.3 for zinc chloride. Afterward, the mixture was dried in an oven with air circulation at 105 °C for 24 h.

The activation of the two conditioned biochars (S2 and S3), along with the biochar precursor (S1), was performed following a procedure similar to that reported by Shuai Guo et al. [23]. The activation process was conducted in a semi-continuous system utilizing a Parr installation equipped with a vertical stainless steel tubular reactor containing a fixed adsorbent bed and a dosing pump. The adsorbent samples were positioned at the bottom in the isothermal zone of the reactor, while the lower and upper regions were filled with ceramic beads. The adsorbents were heated at a rate of 5 °C per minute under a nitrogen atmosphere until they reached a temperature of 550 °C. Subsequently, distilled water was introduced at a volumetric rate of 1 h^−1^ for a duration of 5 h at atmospheric pressure. After cooling overnight under nitrogen circulation, the adsorbents were collected and placed in a desiccator.

### 2.3. Characterization Methods of Grape Seeds

Characterization of grape seeds by chemical compound classes aimed to determine moisture content, ethanol extractables, lignin and carbohydrate content, and ash content. Grape seeds dried at 103 °C for 4 h were finely crushed with a GRINDOMIX GM 200 knife mill, manufactured by Retsch GmbH (Haan, Germany), and sieved to a powder with a particle size ≤ 200 µm.

#### 2.3.1. Determination of Water Content

The analysis was performed by weighing a certain amount of sample and drying it repeatedly in an air-ventilated oven at 105 °C until constant mass. At 5 h intervals, the sample vial was cooled in a desiccator for 30 min and then weighed on an analytical balance. The drying, cooling, and weighing operations were repeated until the difference between two successive weighing results was no more than 0.004 g. The mass loss was reported per 100 g of the original sample.

#### 2.3.2. Determination of Ethanol Extractables

The removal of extractables, such as fats, waxes, sterols, resins, phenolic compounds, etc., was carried out by ethanol extraction in a Soxhlet for 16 h (ASTM E1690_08 Ethanol Extracts of Biomass, ASTM International, 100 Barr Harbor Drive, PO Box C700, West Conshohocken, PA 19428-2959. United States, 2021). The ethanol extractables content was determined with reference to the original sample.

#### 2.3.3. Determination of Lignin Content

The lignin content was determined according to the adapted Standard E-1721 [24]. The principle of this method consists of the precipitation of lignin with a 72% sulfuric acid solution. To obtain conclusive results, we treated 5 g of grape seeds obtained after removal of extractable substances with 75 mL of 72% H_2_SO_4_ solution for 2 h at room temperature to hydrolyze and solubilize carbohydrates. Then, we diluted the sample with water to reduce the sulfuric acid concentration to 3% gr. and heated it under reflux for 4 h. The obtained lignin was filtered, washed with hot water until a neutral pH was reached, then dried at a constant temperature and weighed. The lignin content was determined with reference to the initial sample.

#### 2.3.4. Determination of Carbohydrate Content

The carbohydrate content of the grape seed sample was determined according to the adapted Standard E-1721. Thus, after lignin precipitation, the content of soluble compounds in the 72% H_2_SO_4_ solution (hemicellulose, cellulose, and other carbohydrates such as sugars, etc.) was determined by the difference between the weight of the biomass sample free of ethanol-extractable substances, subjected to treatment with 72% H_2_SO_4_ solution, and the weight of lignin.

#### 2.3.5. Determination of Ash Content

The ash determination was carried out according to the Standard Test Method for Ash in Biomass, ASTM E1755-01 ASTM International, 100 Barr Harbor Drive, PO Box C700, West Conshohocken, PA 19428-2959. United States, 2024. The grape seed sample dried at 105 °C was placed in a crucible, which was previously calcined in an oven and cooled in a desiccator. The crucible with the sample was placed in an oven at 575 °C for 5 h, after which it was cooled and weighed. The difference in weight, expressed as a percentage of the mass of the residue remaining after calcination, represents the ash content of the sample. The result was reported relative to the mass of the sample dried at 105 °C.

### 2.4. Characterization Methods of Adsorbents

The synthesized adsorbent materials were characterized utilizing thermogravimetric analysis, scanning electron microscopy, Fourier transform infrared spectroscopy, and X-ray diffraction. Additionally, the textural properties of the prepared materials were assessed. All the methods used for biochar preparation and characterization are proposed by the authors in accordance with the analysis methods used in similar studies [25,26,27,28,29].

#### 2.4.1. Thermogravimetric Analysis of Adsorbent Precursors

Thermogravimetric analysis (TGA) of adsorbent precursors prepared from grape seeds was performed with a DuPont Thermal Analyst 2000/2100 coupled with a 951 Thermogravimetric Analyzer module in a nitrogen atmosphere in the temperature range of 25–800 °C, with a heating rate of 20 °C/min.

#### 2.4.2. Textural Characteristics of Adsorbent Precursors

The textural characteristics of the adsorbents were determined using a NOVA 2200e gas sorption analyzer (Quantachrome Tools, Boynton Beach, FL, USA). The nitrogen adsorption/desorption isotherm was recorded at 77.35 K, within a relative pressure range (p/p_0_) of 0.005 to 1.0. Data processing was conducted using NovaWin software version 11.03. The specific surface area was calculated using the standard BET (Brunauer–Emmett–Teller) equation, while the total pore volume was estimated based on the desorbed volume at a relative pressure p/p_0_ close to unity, utilizing the Barrett–Joyner–Halenda (BJH) method. The pore size distribution and mesopore volume were derived from the desorption branch of the isotherm by implementing the BJH model. Prior to the adsorption measurements, the samples were degassed in a vacuum at 160 °C for 4 h.

#### 2.4.3. Microstructural Morphologies of Adsorbent Precursors

The microstructural morphologies were analyzed using an ultra-high-resolution Scios 2 HIVAC Dual-Beam FIB-SEM scanning electron microscope (Scios 2 HIVAC Dual-Beam FIB-SEM with ultra-high resolution; Thermo Fisher, Brno, Czech Republic).

#### 2.4.4. X-Ray Diffraction (XRD) of Adsorbent Precursors

For the X-ray diffraction (XRD) study of the analyzed samples, a D8 Advance diffractometer (Bruker-AXS, Karlsruhe, Germany) with a copper anode X-ray tube (Cu-Kα radiation, λ = 1.54 Å), θ-θ configuration, and Bragg–Brentano geometry was used. The measurements were performed in the measurement range of 10–80° (2θ), and the measurement parameters were voltage 40 KV, anode current 40 mA, scan step 0.1°, and scan speed 0.1 °/5 s. The raw files of the measurements were obtained using the XRD Commander software v3.1. For qualitative interpretations, the Diffracplus EVA v.14 software and the PDF-ICDD database were used. Quantitative interpretations using the Rietveld technique were performed in the TOPAS 4.1 software, and peak fitting was performed with the pseudo-Voigt profile function.

#### 2.4.5. Fourier Transform Infrared (FTIR) of Adsorbent Precursors

For the qualitative analysis of the materials, Fourier Transform Infrared (FTIR) spectroscopy was employed to identify the functional groups present in the structure. The equipment used was the Shimadzu IRAffinity-1S spectrophotometer (Shimadzu, Kyoto, Japan), equipped with the GladiATR-10 accessory. Measurements were conducted over a wavelength range of 380 to 2000 cm^−1^, with a spectral resolution of 4 cm^−1^.

#### 2.4.6. Determination of Adsorption Capacity

**Figure 3:**
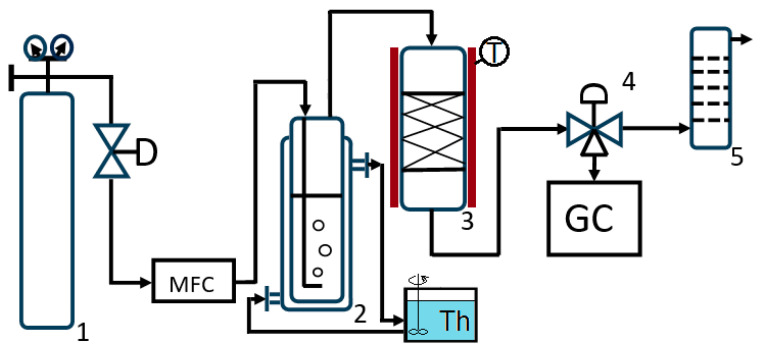
Adsorption plant (1—carrier gas cylinder (nitrogen) with pressure reducer; MFC—mass flow controller; 2—bubbler; Th—thermostat; 3—adsorption reactor; GC—gas chromatograph; 4—three-way valve; GC—gas chromatograph; 5—scrubber).

The adsorption capacity of the prepared adsorbents for toluene was evaluated in a continuous system using a fixed-bed adsorbent, conducted at atmospheric pressure and standard temperature. The experimental setup comprised a pressurized nitrogen gas cylinder outfitted with a pressure reducer, a flow regulator, a rotameter for monitoring gas flow, a bubbler equipped with a thermostatic jacket with a thermal agent, a thermostated absorber featuring an electric heating jacket, and a gas chromatograph process analyzer with a TCD Chromatron GCHF 18.3 (VEB, Berlin, Germany) (Figure 3).

The toluene concentration in the nitrogen carrier gas at the reactor inlet was experimentally determined by continuously weighing the toluene bubbler on a Kern KB2500-2N technical balance (Kern, Baden-Württemberg, Germany), which has a precision of 0.01 g. This process was conducted at a nitrogen flow rate of 265 mL/min while monitoring the evolution of the bubbler’s weight over time. 

## 3. Results and Discussions

### 3.1. Characterization of Biomass Waste

#### Analysis by Classes of Chemical Compounds of Biomass Waste Samples

Table 1 illustrates the chemical class analysis of grape seeds dried at 103 °C for four hours, after which they were finely crushed. As indicated in Table 1, the moisture content is low, enabling pyrolysis processing with a water content below 15%, as suggested by Ahmed et al. [30] The significant carbohydrate and lignin content, coupled with low ash content, are essential factors for the surface, structural, and textural properties of the material, highlighting its substantial potential for producing high biochar yield.

### 3.2. Thermogravimetric Analysis

**Figure 4:**
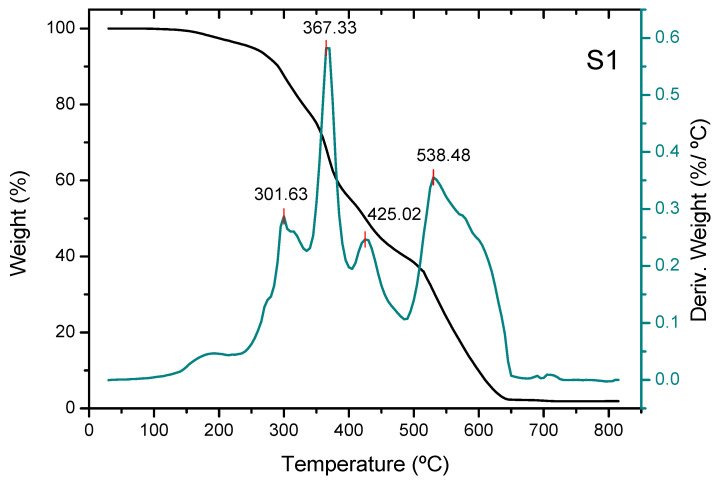
Thermogravimetric analysis of the unconditioned biochar sample, S1 (derivative weight (%/°C) between 0.0 and 0.6).

**Figure 5:**
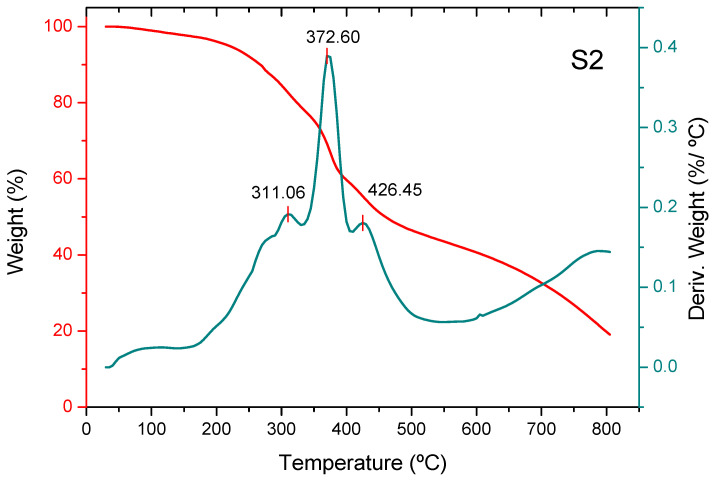
Thermogravimetric analysis of biochar pretreated with phosphoric acid, S2 (derivative weight (%/°C) between 0.0 and 0.4).

**Figure 6:**
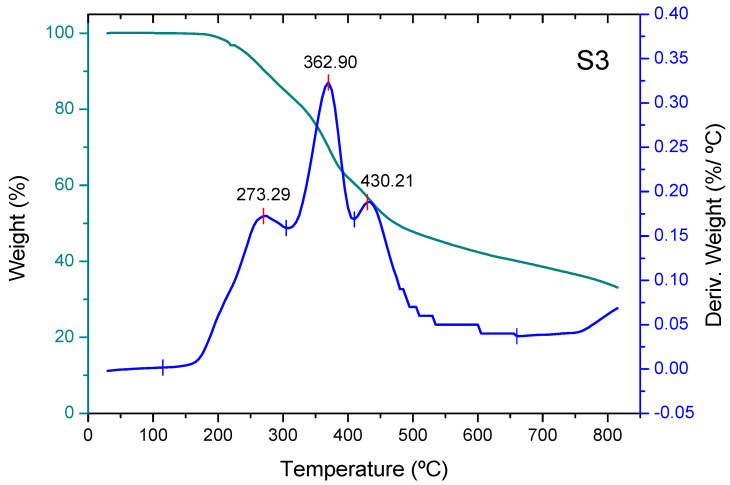
Thermogravimetric analysis of biochar pretreated with zinc chloride, S3 (derivative weight (%/°C) between 0.0 and 0.4).

Thermogravimetric analysis (TGA) reveals three stages in the thermal decomposition process, except for non-activated carbon, where a stage occurs at higher temperatures (nearly 550 °C) (Figure 4). The weight loss of the three adsorbents in the first stage (the devolatilization stage [31]) shows a maximum at approximately 300 °C. The adsorbent added with phosphoric acid shows a maximum increase of approximately 10 °C compared to the non-activated one, and the adsorbent added with zinc chloride shows a maximum decrease of approximately 25 °C compared to the non-activated one. Part of the mass loss in this stage for adsorbent S2 is due to the removal of water resulting from the dehydration of phosphoric acid into polyphosphoric acids [32]. During the second stage, carbonization of structural carbohydrates mainly occurs [33], resulting in a drastic decrease in sample weight. This stage shows a maximum at temperatures around 360–370 °C (Figure 5). The last stage consists of the carbonization of more stable polymers, such as lignin, and shows a maximum at temperatures of around 425–430 °C. The mass loss is much lower than in the previous stage, at a temperature range of 600–900 °C. The mass loss of the unadditive adsorbent in the fourth stage begins at a temperature of around 500 °C, and this is the most important stage. Thus, the total mass loss of the unadditive adsorbent in the temperature range of 20–670 °C approaches around 97% of the initial mass of the non-activated biochar, the total mass loss of the adsorbent pretreated with phosphoric acid in the temperature range of 20–500 °C amounts to approximately 58% of the initial mass of the biochar, and the cumulative mass loss of the biochar pretreated with zinc chloride in the temperature range of 20–500 °C reaches approximately 57% of its initial mass (Figure 6).

The mass loss mechanisms we propose have also been examined in other studies. For instance, S.M. Yakout [34] demonstrated that the reaction of lignocellulose with phosphoric acid occurs through the hydrolysis of glycosidic bonds in lignocellulose and the cleavage of the aryl–ether bond in lignin. These reactions are accompanied by further chemical transformations, which include dehydration, degradation, and condensation. As the acid concentration rises, aromatic condensation reactions can also take place between adjacent molecules, leading to the formation of gaseous products and subsequently reducing the yield of activated carbon. Meanwhile, the BET surface area and total pore volume increase with higher acid concentrations. In another study [35], it was found that increasing the activation temperature (while maintaining the same impregnation rate) results in a decrease in the number of acidic functional groups, while the basic surface groups of carbon tend to increase.

### 3.3. Textural Characteristics

**Figure 7:**
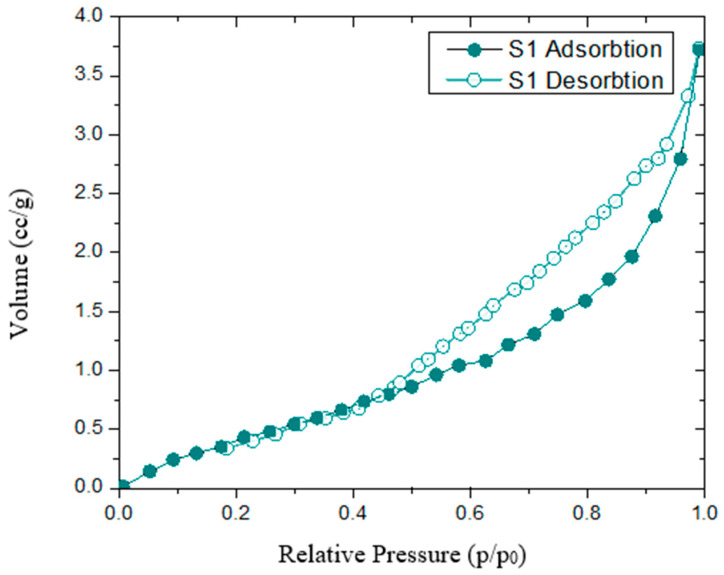
N_2_ adsorption–desorption isotherms for unactivated biochar.

**Figure 8:**
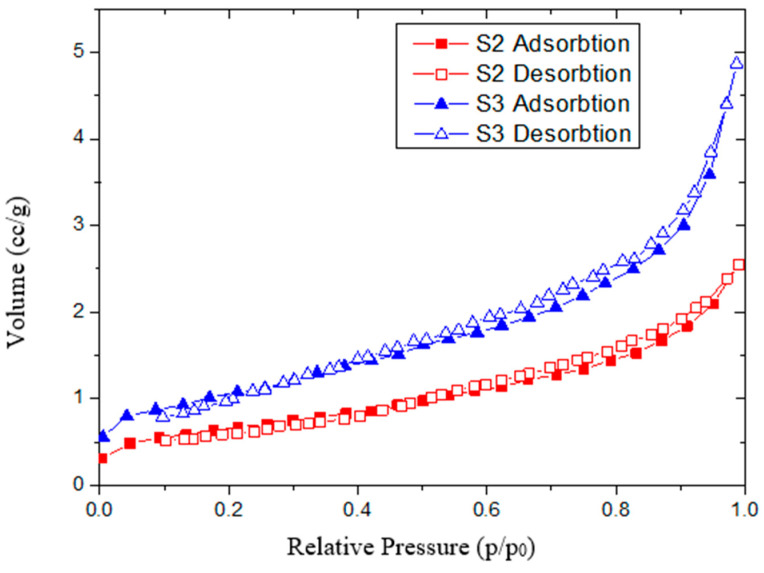
N_2_ adsorption–desorption isotherms for activated biochar.

**Figure 9:**
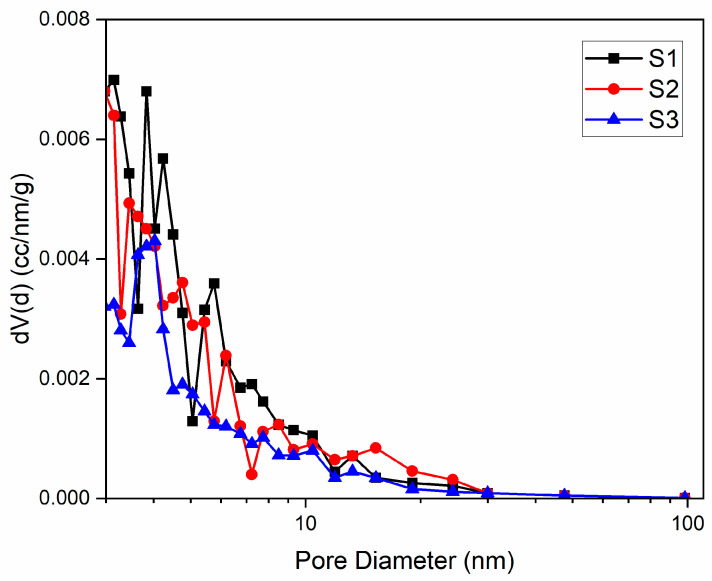
The pore size distribution of activated and unactivated biochar.

Adsorption and desorption isotherms recorded at relative pressures (p/p_0_) from 0.005 to 1.0 were used to assess the textural parameters of biochar adsorbents. Figure 7 and Figure 8 show that all three biochars exhibit type IV isotherms with type H1 hysteresis loops, as classified by IUPAC, which are typical for mesoporous adsorbents [36]. Biochar S1 displays a pronounced desorption branch hysteresis, with greater nitrogen desorption at relative pressures above 0.5, resulting in a wide hysteresis loop that may indicate metastable states [37]. Hysteresis in physical adsorption confirms the presence of mesoporosity [38]. In contrast, S2 and S3 biochars show desorption curves that closely follow the adsorption curves, resulting in narrow hysteresis loops. These isotherms, which overlap at low pressures and show hysteresis only at higher pressures (p/p_0_ > 0.40), are characteristic of steam-activated carbons [39]. The pore size distribution curve for S1 indicates a broad distribution with a significant proportion of macropores (Figure 9). Treatments with phosphoric acid and zinc chloride promote the development of a mesopore network, increasing the pore volume. Table 2 shows that the specific surface area of activated biochar increases by about 20% after treatment, with a higher increase for biochar treated with phosphoric acid (2778 m^2^/g for S2 compared to 2697 m^2^/g for S3). Similar specific surface area values have been reported by other researchers [25,26] for grape seed pyrolysis at comparable temperatures. The increase in porosity is mainly attributed to the enhanced decomposition of tars by phosphoric acid treatment [34]. This change in porosity is accompanied by a decrease in acidic functional groups [35]. The improvement in BET specific surface area following ZnCl_2_ treatment has also been noted by other authors [15]. Gregg et al. further support these findings, demonstrating that porosity increases after biomass carbonization due to the decomposition of volatile matter during pyrolysis [38]. In summary, biochar produced from partially deginified grape seeds and activated at 500 °C with phosphoric acid and zinc chloride shows improved porosity, a larger specific surface area, and greater pore volume compared to non-activated biochar.

### 3.4. SEM Analysis

**Figure 10:**
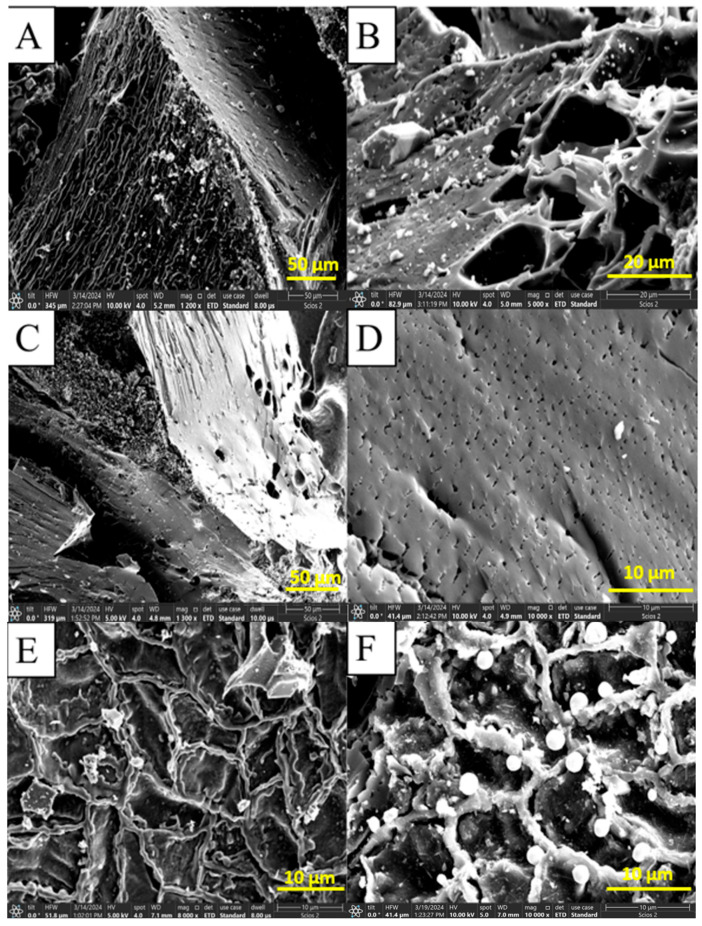
SEM images of the three samples of biochar: (**A**,**B**) biochar samples without pretreatment (S1); (**C**,**D**) biochar treated with phosphoric acid (S2); (**E**,**F**) biochar treated with zinc chloride (S3).

The scanning electron microscopy (SEM) analysis of the biochar samples is presented in Figure 10A,B. The sample without pretreatment appears inhomogeneous, and the enlarged image reveals pores of varying appearances and sizes. 

The scanning electron microscopy analysis of the biochar treated with phosphoric acid (S2), as shown in Figure 10C,D, reveals that the sample displays partial inhomogeneity, with the white regions indicating agglomerated polyphosphoric acid granules. Upon magnification, the image shows pores that are considerably smaller in size compared to those observed in untreated biochar, highlighting a more uniform distribution with varying diameters.

The scanning electron microscopy analysis of the biochar treated with zinc chloride (S3), as evidenced by Figure 10E,F, shows that the sample is partially inhomogeneous, with the white regions likely representing clusters of zinc chloride. Upon magnifying the image, irregularly shaped pores can be observed, which appear to have a relatively homogeneous distribution across different diameters. As Oscar Javier Fonseca-Bermúdez and collaborators point out, the macropores observed in both images improve the accessibility of volatile compounds to the internal microporous structure [33].

The compositions of the three biochars, determined by EDX, are presented in Table 3. In the case of untreated biochar, carbon is present at the highest concentration, making up 92.82%. The other chemical elements identified are present at levels typical of vegetable products. After treatment with phosphoric acid, the biochar undergoes compositional changes. The carbon content drops by nearly 14%, while the phosphorus content nearly doubles, and the oxygen content increases by over 14%.

On the other hand, the composition of biochar treated with zinc chloride is similar to that of the untreated biochar, but zinc is not detected in the treated version. Instead, higher concentrations of chlorine can be found compared to the untreated biochar. Analysis of microscopy images suggests that the zinc present in the biochar is partially hydrolyzed, with light-colored clusters primarily composed of unevenly distributed zinc oxides. These elemental compositions are very different from the applied recipes, emphasizing the migration of certain components that contribute to the inhomogeneities observed in the prepared adsorbents.

### 3.5. XRD Analysis

**Figure 11:**
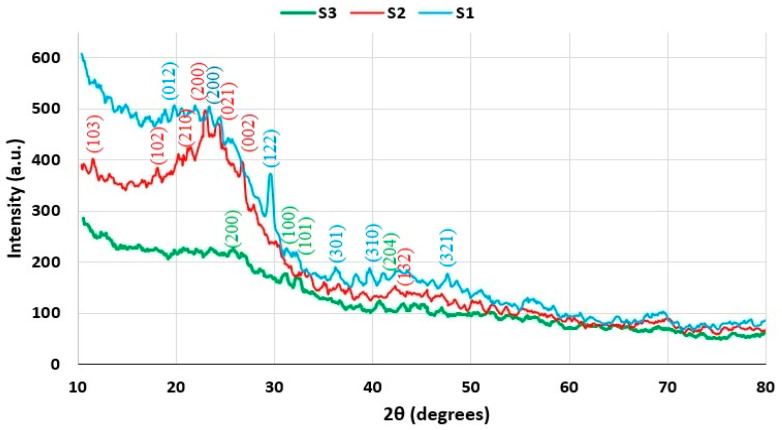
X-ray diffraction pattern for samples S1, S2, and S3.

X-ray diffraction investigations reveal structural differences among the three samples studied: S1, S2, and S3 (Figure 11). While the intensity and width of the peaks vary from one sample to another, the X-ray diffraction spectra exhibit characteristics specific to amorphous materials, as indicated by the broad peak in the range of 15–30° 2θ.

Sample S1 demonstrates characteristic peaks at 2θ values of 23.15°, 29.47°, 36.15°, 39.35°, and 47.42°, which correspond well with the crystalline planes/Miller indices (200), (122), (301), (310), and (321), indicative of lignin biomass structure. Sample S2 exhibits peaks attributed to lignocellulosic biomass at 2θ degrees of 11.36°, 18.04°, 21.24°, 22.84°, 24.00°, and 30.16°, aligning with Miller indices (103), (102), (210), (200), (021), and (208). Additionally, sample S2 features a prominent peak for carbonaceous structures at 26.55°, corresponding to the Miller plane (002). Sample S3, exhibiting the lowest crystallinity, reveals a broad peak within the 15–30° 2θ range, with a maximum at 21.96°, associated with the 200 plane of lignin/biomass. Carbonaceous structure is also detected at 26.55° (plane 002), while zinc oxide appears at 30.64° (plane 100) and 32.05° (plane 101). The distinctive peak for lignin biomass is significantly diminished and broadened in sample S3, indicating a decrease in crystallinity [40,41,42,43,44].

By utilizing the Rietveld method and the Topas 4.1 program, the composition of the three samples was estimated based on the experimental data and the selected structural model, facilitating the determination of the degree of crystallinity. The degree of crystallinity (*X_C_*) was calculated using the following equation:

(1) $$X_{c}=\frac{\sum A_{crys}}{\sum A_{crys}+A_{amorph}}$$

*A_crys_* is the adjusted area of the crystalline phase, and *A_amorph_* is the adjusted area of the amorphous phase.

The presence of carbonaceous structures in samples S2 and S3 is attributed to the lignin carbonization as a result of thermal treatment (Table 4). Additionally, it has been noted that the degree of crystallinity of lignin diminishes due to the depolymerization of lignin, which occurs through homolytic processes that break C-O and C-C covalent bonds during heat treatment.

All three diffractograms show that biochar is mainly amorphous (“turbostratic or hard carbon”), overlain by several mineral phases from the natural ash of the kernels (K, Ca, Mg, Si, P, etc.). Hard carbon is a structure with small-scale in-plane ordering, without interlayer ordering [45,46]. The low value of the specific surface area determined by the BET method, around the average value of 2.5 m^2^/g, confirms the existence of compact “char”-type carbonaceous materials, with few open micro-/mesopores. Moreover, the broad shoulder in the ~20–30° 2θ area is characteristic of turbostratic carbon, with the (002) reflection indicating the presence of disordered “graphite-like” structures.

The XRD spectrum for sample S1 (blue) indicates a higher amorphous background and more mineral peaks and probably higher ash content, with mineral phases remaining well crystallized. The XRD spectrum for sample S2 (red) shows fewer visible mineral phases; the (002) plane of the carbon is similar to that of the amorphous carbonaceous structure, with the difference being lower ash content.

When treated with H_3_PO_4_, dehydration can also occur, leading to the formation of phosphate bridges in the solid phase; in the presence of Ca/K from the biomass, carbonates tend to transform into phosphates or pyrophosphates, and we can notice this transformation in the XRD spectra, which show the diminution of the calcite peaks (~29.4°). This “glassy” phosphate phase can even block the pores, explaining the low BET specific surface value [47].

The XRD spectrum for sample S3 (green) shows a lower overall intensity and very few sharp peaks, indicating the lowest ash content and a higher degree of disorder of the carbonaceous structures, respectively.

Moreover, the XRD analysis results correlate with the BET analysis results, typical of primary, amorphous, non-activated biochar structures, with pores probably blocked by ashes or tars. Although two activating “pretreatments” were performed, the surface area of the prepared materials remained at low values. An explanation of the results obtained in this research step could be that the activation conditions were not optimal to obtain an increased effective porosity.

### 3.6. FTIR Analysis

**Figure 12:**
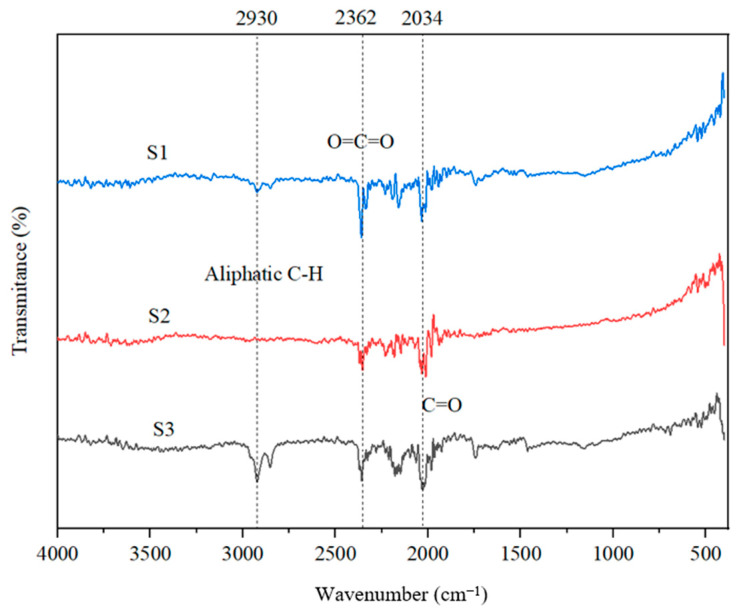
FTIR spectra of absorbents S1, S2, and S3.

Figure 12 presents the FTIR spectra of the three biochar samples. For the sample treated with zinc chloride (S3), a notable band at 2928 cm^−1^ corresponds to the stretching vibrations of the C-H bonds in aliphatic structures. This suggests that the condensation process catalyzed by ZnCl_2_ facilitates electrophilic substitution reactions involving aliphatic groups, which likely result from the degradation of specific compounds found in grape seeds. Furthermore, the band at 2364 cm^−1^ indicates the presence of CO_2_, a compound that may arise from the decomposition of certain carboxylic components present in the biomass or by the adsorption of CO_2_ from air. The vibrations observed at 2034 cm^−1^ are linked to C=O bonds in carbonyl structures, as well as to carboxyl groups. The presence of zinc oxide facilitates the formation of carboxylate metal complexes. In the sample treated with phosphoric acid, the intensity of the vibrations at 2034 cm^−1^ decreases due to the blocking of carboxyl sites. On the other hand, in the untreated sample, the vibrations at this wavenumber are attributed to oxygenated carboxyl, hydroxyl, or ether groups [48].

### 3.7. Determination of Toluene Adsorption Capacity on Additivated Adsorbents

Prior to starting the adsorption experiments, the biochar was placed in an oven at 130 °C for 24 h and then stored in a desiccator until it was ready to be loaded into the adsorption reactor. The concentration of toluene in the nitrogen carrier gas was determined experimentally using the gravimetric method. This involved periodically weighing a bubbler containing toluene into which nitrogen was bubbled at atmospheric pressure and at a predetermined flow rate, according to the experimental program.

**Figure 13:**
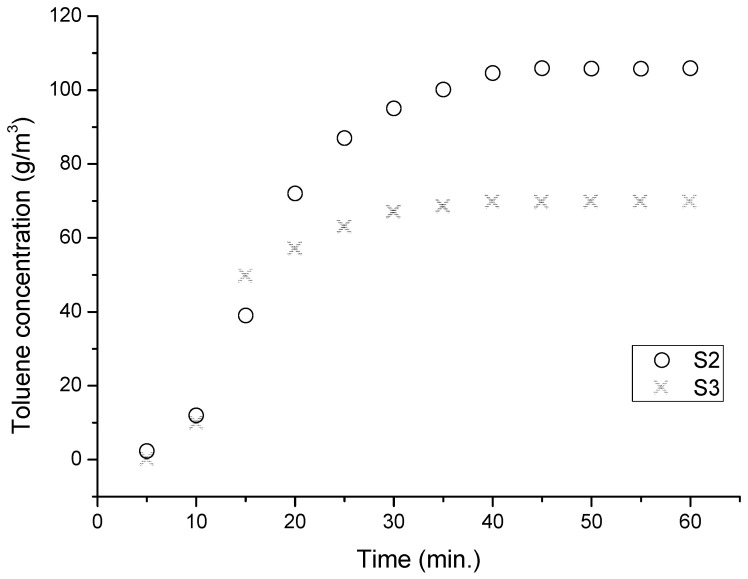
Variation in toluene concentration in the nitrogen flow discharged from the adsorber with contact time in the case of adsorption on biochars treated with phosphoric acid (S2) and ZnCl_2_ (S3) at 25 °C and atmospheric pressure.

The residual concentration of toluene in the nitrogen flow exiting the adsorption reactor was measured using a process chromatograph. This setup included a chromatographic column that was 2 m long with an inner diameter of 2 mm, containing 1 m of molecular sieves 10 Å as the stationary phase and 1 m of activated biochar. The nitrogen used as the carrier gas had a purity of 6.0, and the operation was conducted at a constant pressure of 800 kPa. The thermal conductivity detector (TCD) was operated with a sensitivity of 16× and a current of 180 µA. Figure 13 illustrates the variation in toluene concentration in the nitrogen flow discharged from the bubbler, corresponding to the contact time for the two types of biochar, at a nitrogen flow rate of 200 mL/min.

The adsorption capacity of toluene on the tested adsorbents was calculated by integrating the area up to the point of penetration of the adsorbent layer using the method presented by Liangliang Deng et al. [49]

Analysis of the adsorption curve for toluene on biochar pretreated with ZnCl_2_ indicates that after 14 min of exposure, the adsorbent becomes approximately 50% saturated. Full saturation of the adsorbent occurs roughly 35 min after exposure to the pollutant, with a maximum adsorption capacity determined to be 0.11 cm^3^ of toluene per gram of adsorbent.

In contrast, the adsorption curve for toluene on biochar pretreated with phosphoric acid shows that after 17 min of exposure, the adsorbent is also saturated at around 50%. The carrier gas flow rate is optimal, and the adsorption curve demonstrates effective prevention of penetration into the adsorbent layer. Full saturation occurs approximately 42 min into the experiment, and from the adsorption curve, we can observe that the maximum adsorption capacity for this adsorbent is found to be 0.14 cm^3^ of toluene per gram of adsorbent.

### 3.8. Discussion

Partial delignification of grape seed biochar by depolymerization by nucleophilic attack with sodium sulfide at relatively low temperatures did not allow stabilization of the porous structure. Thus, the specific surface area of the delignified biochar was lower than that of the biochar conditioned with phosphoric acid or zinc chloride. It is likely that the tar formed following partial delignification cannot be completely removed by washing with water and drying in an air circulator.

The acidic additives used in the conditioning of the biochar influenced the carbonization of the tar remaining in the pores by promoting condensation reactions, contributing to their unblocking. The significant mass losses observed in the S1 biochar, as indicated by the TG analysis, are probably due to the partial depolymerization reactions that reduced the molar mass of lignin, favoring lignin cracking reactions during the grape seed conditioning step. In addition, conditioning of activated biochar with phosphoric acid and zinc chloride promoted the recondensation of the undissolved lignin fraction, resulting in a relatively stable porous structure. This improvement is probably due to the catalytic properties of these agents, which facilitate both the advanced condensation of tar to carbonized structures and the cracking of lower-molecular-weight macrocompounds to lighter compounds, which can be removed by steam stripping [34,35].

The adsorption–desorption isotherm with liquid nitrogen, obtained from the textural analysis, indicated that after conditioning of grape seeds, the desorption curve closely overlapped the adsorption curve. This trend was particularly pronounced in the samples conditioned with phosphoric acid. The low value of the hysteresis for the two curves indicates the presence of a stable microporous structure of the two biochars, while the high value of the hysteresis for the S1 biochar is due to a predominantly mesoporous structure [50]. This improvement of the porous structure after treatment with acid catalysts is also highlighted by XRD analysis. Thus, the decrease in lignin content accompanied by the increase in partially crystalline carbonaceous structures content after conditioning with additives demonstrates the catalytic activity of these additives in condensation processes. At the same time, the high degree of crystallinity of the partially crystalline carbonaceous structures obtained is also due to the advanced condensation catalytic activity of the two additives used [40,44].

SEM analysis of the biochar showed an improved pore distribution after activation with phosphoric acid and zinc chloride. The analysis revealed an uneven agglomeration of polyphosphoric acid clusters on the pore surface, while zinc-based clusters were more evenly distributed. Compositional analysis of the three biochars, performed by EDX, indicated that the phosphoric acid-treated biochar showed an increase in phosphorus content. In contrast, in the zinc chloride-treated biochar, the observed increase in chlorine content was not accompanied by a corresponding increase in zinc content. This can be attributed to the hydrolysis of zinc chloride and the subsequent migration of Zn ions.

The adsorption capacity values of toluene on the additivated adsorbents are similar to those reported in the literature. Thus, Lei Xu, Yonghong Li, and collaborators obtained a toluene adsorption capacity on an ultra-stable Y zeolite of 0.18 g/g, and Reem A. Essa and collaborators obtained a methylene blue adsorption capacity on a modified biochar of 118.8 mg/g [51,52]. The phosphoric acid-pretreated biochar demonstrated superior performance compared to the zinc chloride-treated one. Despite the incomplete pore formation and relatively small pore volumes for the undoped adsorbent, the adsorption capacity obtained was close to that of the doped biochar. The relatively high adsorption capacity of the three adsorbents relative to the small pore volume values can be attributed to strong interactions between toluene and these adsorbents obtained by partial extraction of lignin by nucleophilic attack.

## 4. Conclusions

This study shows that the method of preparing activated biochar by partial delignification of grape seeds is an alternative to the methods applied so far. Thus, the removal of the lignin fraction with a lower molecular weight by depolymerization and solubilization reduces the tar content in the pores of the biochar, and thermal treatment at lower temperatures improves the porous structure. Conditioning the partially delignified biochar by treating it with a Brønsted or Lewis acid improves the porous structure and the performance of the obtained biochar for the adsorption of atmospheric pollutants such as toluene.

## Figures and Tables

**Table 1:** Chemical composition of the dried grape seed sample.

Components	Value
Moisture, wt.%	2.16
Ethanol extractables, wt.%	17.28
Lignin, wt.%	31.69
Carbohydrates, wt.%	48.87
Ash, wt.%	3.61

**Table 2:** Textural characteristics for grape seed biochar adsorbents. The standard deviation did not exceed 5%.

No.	Adsorbant	Specific Surface Area (m^2^/g)	Total Pore Volume (cm^3^/g)	Average Pore Diameter (nm)
1.	S1	2.187	0.0041	6.535
2.	S2	2.778	0.0057	5.754
3.	S3	2.697	0.0054	5.833

**Table 3:** Atomic percentages of each element in biochar samples.

% Weigh	Unconditioned and Unactivated Biochar	Biochar Treated with Phosphoric Acid	Biochar Treated with Zinc Chloride
C	92.82%	79.07%	94.34%
O	-	14.33%	-
Mg	1.32%	0.33%	0.23%
P	3.42%	6.44%	0.96%
S	0.08%	-	0.16%
Cl	0.05%	-	2.5%
Ca	2.30%	0.13%	1.8%

**Table 4:** Estimated composition (wt.%) and degree of crystallinity of samples S1, S2, and S3.

Sample	Lignin-Based Biomass,%	Carbonaceous Structures,%	Zinc Oxide,%	*X_c_* % Lignin	*X_c_* % Graphite	*X_c_* % Zinc Oxide
S1	100	-	-	33.06	-	-
S2	69.20	30.80	-	29.36	94.70	-
S3	35.33	49.07	15.61	18.09	92.67	89.24

## Data Availability

The original contributions presented in this study are included in the article. Further inquiries can be directed to the corresponding author.
